# The Use of Magnetic Resonance Spectroscopy and Magnetic Resonance Imaging in Alcohol Research

**Published:** 2008

**Authors:** Bonnie J. Nagel, Christopher D. Kroenke

**Keywords:** Alcoholism, alcohol dependence, alcohol and other drugs effects and consequences, brain function, brain structure, neuropathology, neuroimaging, magnetic resonance imaging (MRI), functional magnetic resonance imaging (fMRI), animal studies, human studies

The recent emergence of magnetic resonance (MR)-based neuroimaging techniques has dramatically improved researchers’ ability to understand the neuropathology of alcoholism. These techniques range from those that directly monitor the metabolism and the biochemical and physiological effects (i.e., the pharmacodynamics) of alcohol within the brain to techniques that examine the impact of heavy alcohol use on brain structure and function.

In general, MR-based techniques measure electromagnetic signals (the same type of signals detected by a radio antenna) generated by nuclei of endogenous molecules in the body of a person placed in a powerful magnet field. When influenced by a magnet, tissue itself transiently becomes magnetic. In part, this is because of the properties of atomic nuclei. Different MR-based techniques have been developed to utilize nuclear magnetism induced in tissue to generate images of internal structure. The most commonly used MR imaging (MRI) techniques rely on signals derived from hydrogen nuclei in water, which is by far the most concentrated molecular species in the body. The physical properties of water molecules vary from one region of tissue to another, and this influences the nuclear magnetism generated by water hydrogen nuclei. As a result, MRI can differentiate regions in soft tissue at a high level of detail. A second approach—MR spectroscopy (MRS)—uses the same strategy to detect electromagnetic signals, but they are derived from nuclei of atoms (hydrogen as well as some other atoms) on molecules other than water, such as lipids, amino acids, or even alcohol (i.e., ethanol). The resulting data on the molecule(s) under investigation can provide detailed information about the metabolic activity of various tissues, including the brain. The main advantage of MR-based techniques is that they do not expose the subject to radioactive tracers and therefore can be used repeatedly in the same subject, allowing researchers to track metabolic or structural changes over time.

This article briefly summarizes how these techniques may be used to characterize the effects of alcohol dependence on the brain.

## Direct Measurement of Alcohol in the Brain

**Figure 4:**
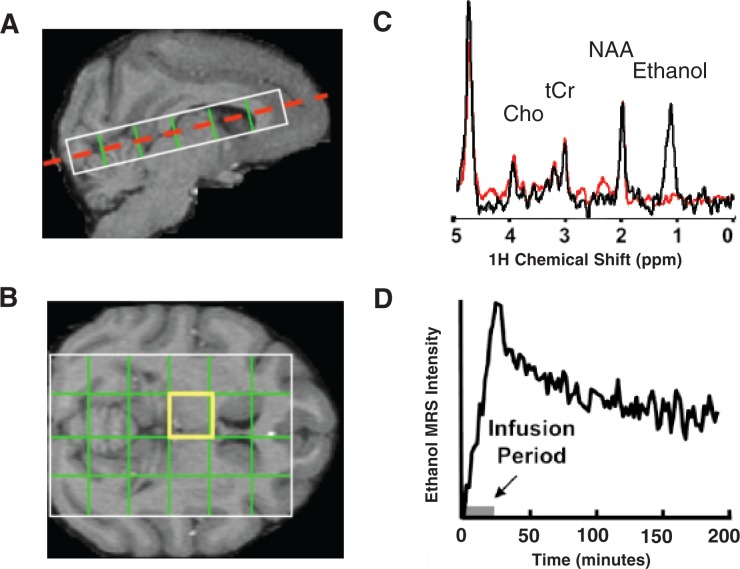
Magnetic resonance spectroscopy (MRS) of ethanol in the nonhuman primate brain. **A)** MRS data acquired from a rhesus macaque over the course of a 2-g/kg intravenous infusion of alcohol. The image shows a lengthwise cut through the brain, with the white rectangle delineating the area that was used for the analysis. **B)** Specifically, spectra were acquired from each of the 24 regions delineated by the grid, which is projected on a horizontal image of the brain at the position indicated by the red dashed line in panel A. **C)** An example of an MRS spectrum obtained from the highlighted (yellow) brain region in B obtained prior to alcohol infusion (red trace) and again following alcohol infusion (black trace). The spectrum shows the ethanol peak as well as peaks for other endogenous compounds, such as *N*-acetylaspartate (NAA), choline-containing compounds (Cho), and creatine (tCr). **D)** The alcohol signal is quantified versus time.

As indicated above, MRS is the most direct MR-based technique for studying alcohol in the brain. This approach has been used to characterize alcohol pharmacodynamics in rodents (Adalsteinsson et al. 2006), humans (Hetherington et al. 1999), and nonhuman primates (see figure 4). However, it is unclear whether this technique can measure ethanol concentrations in the brain accurately because in several quantitative studies, MRS-based estimates of alcohol concentrations in the brain were reported to be lower than expected, based on blood alcohol concentration measurements (Chiu et al. 2004; Kaufman et al. 1994, 1996; Moxon et al. 1991). To explain this observation, Moxon and colleagues (1991) have argued that the hydrogen nuclei of some of the ethanol molecules (i.e., of those that are bound to membranes) possess certain characteristics^1^ that make them undetectable by in vivo MRS. This phenomenon may be relevant for alcoholism research because some evidence suggests that the amplitude of the MRS signal for alcohol that can be observed following a given alcohol dose changes with repeated alcohol exposure (Govendaraju et al. 1997; Moxon et al. 1991) and that this change potentially is related to the development of tolerance (Kaufman et al. 1994, 1996). To clarify the potential link between changes in alcohol MRS intensity and alcohol exposure, it is therefore important to determine whether alcohol truly is partially “invisible” to MRS in the brain (Chiu et al. 2004) and whether brain alcohol concentrations may be accurately measured by MRS if the relevant characteristics of the hydrogen nuclei are carefully determined (Hetherington et al. 1999; Sammi et al. 2000).

The effects of chronic alcohol exposure on the brain and its neurochemistry also can be assessed through MRS measurements of endogenous compounds naturally produced in the body. One of these is a compound called *N*-acetylaspartate (NAA), which is one of the most abundant molecules in neurons and usually provides a large signal in brain MRS measurements (see figure 4C) (Mason et al. 2005, 2006). NAA levels are reduced in numerous neuropathological conditions. According to one report, chronic heavy drinkers also exhibit reduced intensity of the NAA signal compared with control subjects (Mason et al. 2005), with larger effects seen in females than in males. Although this observation is consistent with several potential explanations (Mason et al. 2006), one popular interpretation of reduced NAA levels in drinkers is that it reflects some form of neuronal loss or pathology.

## Assessing Structural Changes Associated With Alcohol Use

**Figure 5:**
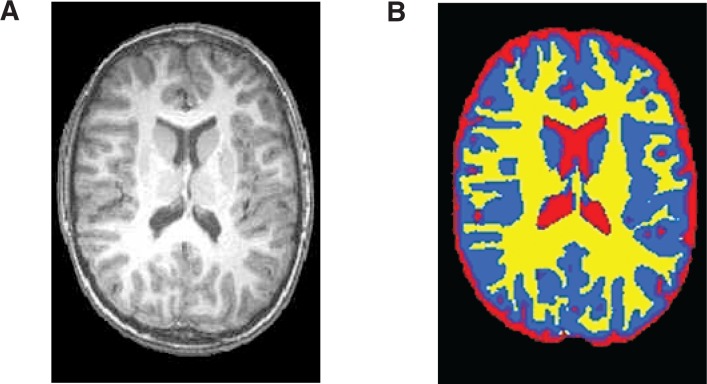
Example of an MRI image of the brain, showing gray matter (blue), white matter (yellow), and cerebral spinal fluid (red). NOTE: The image is a segmented skull-stripped T1-weighted anatomical image. This automated segmentation was performed using Oxford Centre for Function Imaging of the Brain’s (FMRIB) Automated Segmentation Tool (FAST).

It is well known that chronic alcohol use is associated with gross anatomical changes in the brain. Structural MRI analyses in particular have greatly enhanced our understanding of these alcohol-related changes. Based on differences in certain properties (i.e., spin relaxation properties) of water molecules in various types of brain tissue, researchers can classify individual volume elements (i.e., voxels) on the MRI images into gray matter, white matter, and cerebral spinal fluid (see figure 5). Using these methods, several studies have revealed alcohol-related reductions in gross brain tissue volumes (Kril and Halliday 1999). In addition, the high resolution of MRI has facilitated the measurement of smaller structures in the brain, and studies have shown reductions in the volume of various brain structures, including the hippocampus (Agartz et al. 1999; Beresford et al. 2006), corpus callosum (Hommer et al. 1996; Pfefferbaum et al. 1996), striatum (Sullivan et al. 2005), and cerebellum (Shear et al. 1996), in people with alcohol use disorders. Because MRI analyses can be performed repeatedly in the same subject, the technique allows for longitudinal followup of alcohol-dependent people after treatment. Such studies have suggested that structural recovery in the brain may be possible in people achieving sustained abstinence (Cardenas et al. 2007; Shear et al. 1994).

An additional MRI-based technique, termed diffusion tensor imaging (DTI), allows investigators to study brain pathology on a microstructural scale. This technique exploits the passive movement (i.e., diffusion) of water molecules within a tissue or structure. For example, many neurons have one long extension (i.e., the axon) that connects to other nerve cells and transmits signals to them. This axon typically is surrounded by a sheath made up of a molecule called myelin. Furthermore, the myelin-covered axons of several nerve cells may be held together in axon bundles. Because the myelin gives these bundles a whitish appearance, brain areas containing many of these bundles also are referred to as white matter (as opposed to gray matter, which is made up of nerve cell bodies). In healthy white matter, myelinated axon bundles selectively restrict water diffusion, so that the water molecules tend to move along the white matter tracts but not in a perpendicular direction. As a result, diffusion is orientation dependent, or anisotropic. DTI measurements have identified reduced diffusion anisotropy within the frontal white matter of chronic alcoholics (Harris et al. 2008; Pfefferbaum et al. 2005, 2006), which is interpreted as a manifestation of alcohol-related white matter damage. This interpretation is further supported by findings that deficits in diffusion anisotropy are associated with impairments in working memory (Pfefferbaum et al. 2000).

## Functional MRI Studies Related to Alcohol Dependence

Functional MRI (fMRI) is a powerful tool that allows researchers to assess blood flow, and thereby brain function, in a specific brain region. In general, blood flow is increased in brain regions that are active at a given time and decreased in inactive regions or areas affected by illness or damage. One way of assessing blood flow is by using positron emission tomography (PET), which uses radioactive tracer molecules to track blood flow. (For more information, see the article by Thanos et al., pp. 233–237.) However, the use of radioactive compounds is an obvious disadvantage of that approach, which can be avoided by fMRI. It is based on the observation that blood supplies oxygen to active neurons at a greater rate than to inactive neurons. The increased delivery of oxygen to a specific brain region leads to a magnetic signal variation that can be detected using an MRI scanner. By taking rapid sequences of images and tracking these variations, researchers can examine brain functioning during a variety of cognitive and behavioral tests.

fMRI has furthered alcohol research by allowing investigation of the neural circuits that are impacted by alcohol use. For example, fMRI has revealed abnormal responses in the frontal lobe during verbal and spatial working memory tasks in alcoholics (Desmond et al. 2003; Tapert et al. 2001). In addition, it has enriched researchers’ understanding of the course of alcohol abuse, dependence, and recovery by allowing repeated studies at various points during the course of the disease. However, beyond detecting such functional abnormalities in brain response associated with cognitive tasks, fMRI has tremendously helped scientists identify the neural substrates of alcohol dependence itself. Thus, fMRI studies have elucidated the neural substrates of alcohol craving (Park et al. 2007). Another fMRI study of alcohol cue–related reactivity demonstrated increased reward-based activity in response to alcohol cues in a brain region called the ventral striatum, whereas non–alcohol-related rewards elicited a reduced brain response (Wrase et al. 2007). Abnormal brain responses in these regions have been associated with susceptibility to relapse (Sinha et al. 2007), and pharmacological treatments of alcoholism have shown to reduce abnormalities in alcohol cue–related responding in the ventral striatum (Myrick et al. 2008).

## Conclusions

Different MR-based technologies have allowed researchers to monitor alcohol levels in the brain, identify alcohol-induced structural changes in the brain, and study the impact of alcohol on brain function. To date, most of these studies have been conducted in human subjects. As described in the following article by Boudreau and colleagues, recent technological advances have allowed the application of these approaches also for studying various aspects of alcohol dependence in mouse models.

## Footnotes

1 Moxon and colleagues (1991) suggested that the spin–spin relaxation time constants (T2) of the 1H nuclei of membrane-bound ethanol is so short that these nuclei cannot be detected by MRS.
